# Chlamydia and gonorrhoea infections in young Kenyan HIV-negative cisgender men who have sex with men and transgender women: a multicentre cohort study

**DOI:** 10.1136/bmjopen-2025-098916

**Published:** 2025-08-01

**Authors:** Eduard J Sanders, Elizabeth Wahome, Fredrick Otieno, Joshua Kimani, Alice Bentzer, Duncan Okall, Joseph Nzioka, Evans Gichuru, Elise M van der Elst, Robert C Bailey, Supriya D Mehta, Susan M Graham

**Affiliations:** 1 Implementation Science Division, The Aurum Institute for Health Research, Parktown, South Africa; 2 Dunn School of Pathology, University of Oxford, Oxford, UK; 3 Division of Medical Sciences, University of Oxford, Oxford, UK; 4 UNIM Research and Training Centre, Nyanza Reproductive Health Society, Kisumu, Kenya; 5 University of Nairobi, Nairobi, Kenya; 6 Univeristy of Lund, Lund, Sweden; 7 Nyanza Reproductive Health Society, Kisumu, Kenya; 8 KEMRI-Wellcome Trust Research Programme, Kilifi, Kenya; 9 Faculty of Social Sciences, Utrecht University, Utrecht, The Netherlands; 10 School of Public Health, University of Illinois at Chicago, Chicago, Illinois, USA; 11 Department of Medicine, University of Washington, Seattle, Washington, USA

**Keywords:** INFECTIOUS DISEASES, Epidemiology, Sexually Transmitted Disease

## Abstract

**Abstract:**

**Objectives:**

To assess the prevalence, incidence and factors associated with *Chlamydia trachomatis* (CT) and *Neisseria gonorrhoeae* (NG) infection among HIV-negative men who have sex with men (MSM) and transgender women (TGW) in Kenya.

**Design:**

Prospective cohort.

**Setting:**

Kisumu, Nairobi and coastal Kenya.

**Participants:**

650 young adult participants (570 MSM and 80 TGW) recruited at three research clinics. Inclusion criteria were HIV-negative status, age 18–29 years, assigned male sex at birth, identification as cisgender male or transgender female and reported anal intercourse with a man in the past 3 months.

**Primary and secondary outcome measures:**

Urine, rectal and oropharyngeal samples were tested for CT/NG infection at two different time points (∼6 months apart), using nucleic acid amplification. We compared CT/NG prevalence and incidence in MSM versus TGW and used Poisson regression to compare risk for each group after adjustment for other correlates of prevalent and incident CT/NG infection.

**Results:**

Prevalence of CT/NG infection at any anatomic site was 15.8% and 27.5% in MSM and TGW, respectively (p=0.009). CT/NG incidence was 27.2 (95% CI 21.3 to 34.7) and 24.5 (95% CI 12.3 to 49.0) per 100 person years for MSM and TGW, respectively (p=0.784). In multivariable analysis, there was no difference in prevalence or incidence by gender identity. Baseline CT/NG infection was more prevalent among TGW (adjusted prevalence ratio 1.61, 95% CI 0.99 to 2.62). Incident CT/NG infection was increased among participants with baseline CT/NG infection (adjusted incidence rate ratio (aIRR) 3.14, 95% CI 1.94 to 5.07) and self-reported pre-exposure prophylaxis use (aIRR 1.75, 95% CI 1.04 to 2.93).

**Conclusion:**

Despite higher prevalence of CT/NG infections among TGW at baseline, there were no differences in CT/NG prevalence and incidence between TGW and MSM, after adjustment for potential confounders. Improved condom use, effective partner notification and treatment, and new strategies such as doxycycline post-exposure prophylaxis are needed to reduce CT/NG infections in both MSM and TGW in settings where regular testing is not possible.

**STRENGTHS AND LIMITATIONS OF THIS STUDY**

- Multicentre cohort study of young men who have sex with men and transgender women in Kisumu, Nairobi and coastal Kenya employing iris-scanning for identification to prevent co-enrolment at another location.
- Nucleic acid amplification testing (NAAT) employed to diagnose both prevalent and incident *Chlamydia trachomatis* (CT) and *Neisseria gonorrhoeae* (NG) infections undetectable by standard Kenyan national guidelines.
- Inclusion criteria limited participation to HIV-negative adults aged 18–29, precluding insights into younger adolescents and adults 30 years and older.
- Only a limited number of oropharyngeal samples were obtained and tested due to limited funding, making our oropharyngeal CT/NG prevalence and incidence estimates uncertain.
- No record was kept on partners’ receipt of expedited partner treatment.

## Introduction

 Bacterial sexually transmitted infections (STI) caused by *Neisseria gonorrhoeae* (NG) and *Chlamydia trachomatis* (CT) are highly prevalent and a major cause of morbidity among cisgender men who have sex with men (MSM) and transgender women (TGW). In sub-Saharan Africa (sSA), published prevalence estimates among MSM range from 3.0–11.5% for NG and 10.0–14.5% for CT.^1^,^4^ Without timely screening and treatment, these STIs lead to a range of complications among vulnerable populations and their sexual partners and increase HIV acquisition risk. Indeed, modelling studies estimate that 10.2% of all HIV infections among MSM are attributable to NG and CT infections alone.^5^

There is a paucity of evidence about bacterial STI among TGW compared with MSM in sSA, with many studies either excluding TGW or not distinguishing TGW from MSM in studies of individuals assigned male at birth.^16^,^8^ In a cross-sectional study of rectal and urethral NG and CT among 612 MSM and 70 TGW in Nairobi, rectal NG prevalence was higher among TGW than MSM (21% vs 12%, p=0.06), but there was no difference in rectal CT or urethral NG or CT prevalence.^9^ In a recent study of self-reported sexual behaviours among TGW and MSM at enrolment into a multicentre cohort study, TGW were more likely than MSM to report receptive anal intercourse (RAI) and engaging in group sex, and to have more male sex partners and more male transactional sex partners.^10^

We hypothesised that MSM and TGW living in the same communities in Kenya would have different CT/NG prevalence and acquisition risks as a result of differences in sexual behaviours. We therefore assessed the prevalence, incidence and correlates of CT/NG infection among young HIV-negative MSM and TGW in a multicentre cohort study on HIV acquisition risk in Kenya.

## Methods

Data derived from a multicentre HIV-1 incidence study conducted at research centres located in three different geographical locations in Kenya: Kisumu, Nairobi and coastal Kenya (two sites, Mtwapa and Malindi). Criteria for study eligibility included: age 18–29 years, HIV negative, assigned male sex at birth, identifies as cisgender male or transgender female, reported anal intercourse with a man in the past 3 months, resident near one of the study sites and willing to provide informed consent. Enrolment started in September 2019 and follow-up continued through December 2022, with quarterly visits except during COVID-related clinic closures.^11^

### Study procedures

All participants were tested for CT/NG infections at two time points, approximately 6 months apart, in the period October 2021–December 2022. All participants provided urine samples and had rectal swabs collected by a research clinician. Due to limited funding, oropharyngeal swabs were only collected from a random sample of approximately one third of participants at baseline and follow-up. Detection of CT/NG in these specimens was performed using nucleic acid amplification testing (NAAT) (Cepheid, Sunnyvale, California, USA).

At enrolment and all subsequent study visits, participants completed an audio computer-assisted self-interview in English, Swahili or Dholuo for sociodemographics, sexual behaviours, depressive symptoms (Patient Health Questionnaire 9 (PHQ-9)), alcohol use (Alcohol Use Disorder Identification Test (AUDIT)), use of substances other than alcohol and tobacco (Drug Abuse Screening Test 10 (DAST-10)) and use of pre-exposure prophylaxis (PrEP).

Participants received 650 Kenyan shillings (US$5.6) for scheduled follow-up study visits. All participants had a biometric iris scan taken to verify identity during visits and avoid duplicate enrolments across study sites.

Participants diagnosed with CT/NG infection were contacted and treated according to Kenyan STI treatment guidelines, which recommended oral treatment with cefixime 400 mg directly observed for NG infection only, doxycycline 100 mg two times per day for 7 days for CT infection only, and cefixime 400 mg and azithromycin 500 g directly observed for combined CT/NG infection. While treatment with cefixime and azithromycin was directly observed, 7-day courses of doxycycline were provided to participants after education and instruction by research clinicians, without follow-up to confirm treatment completion. Expedited partner treatment (EPT; ie, medication provided to a patient diagnosed with an STI to give to their partners) was offered to participants in ongoing partnerships, in accordance with Kenyan guidelines.^12^

### Measures

#### Primary outcomes

**Figure 1:**
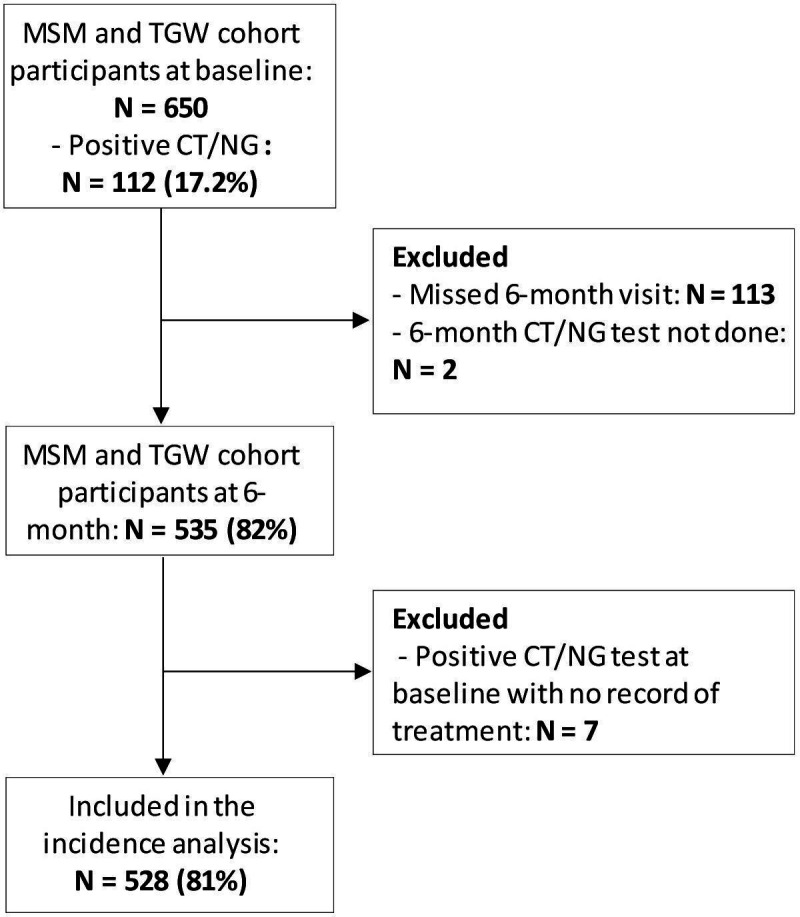
Flow chart of HIV-negative cisgender men who have sex with men and transgender women assessed for chlamydia and/or gonorrhoea infections at baseline and follow-up, Kenya, 2019–2022. CT, Chlamydia trachomatis; MSM, men who have sex with men; NG, Neisseria gonorrhoeae; TGW, transgender women.

Prevalent CT/NG infection was defined as a positive CT and/or NG infection at any site (ie, urethral, rectal or oropharyngeal) diagnosed by NAAT at baseline. Incident CT/NG infection was defined as a positive CT and/or NG infection at any site (ie, urethral, rectal or oropharyngeal) diagnosed by NAAT during follow-up among participants who were negative at baseline or were treated for prevalent infection at baseline. Figure 1 presents a flow chart of participants tested at baseline and follow-up, as well as the sample for the incidence analysis.

#### Secondary outcomes

HIV infection: Individuals were followed quarterly, with HIV counselling and testing at each study visit. The estimated date of HIV infection (ie, seroconversion) was calculated as described previously.^13^ Syphilis testing was not conducted in this study.

#### Covariates

Data collected at enrolment included age, gender identity, ever married to a woman, education, employment status and religion. We also obtained at each 3-monthly visit: condom use during last sex, gender identity of last sexual partner (male or female), sexual partner type (regular, casual, paying or paid (transactional) or missing responses) for the last male and last female partner reported in the past 3 months, and AUDIT score (<8 or ≥8). Participants were asked about the following behaviours in the past 3 months: number of male and female partners (none, one, two or more), RAI (yes, no), insertive anal intercourse (IAI: yes, no), condom use for RAI (yes, no, no RAI), condom use for IAI (yes, no, no IAI), receiving payment for sex, paying for sex and daily oral PrEP use. The following information was collected at enrolment and updated half-yearly: moderate-to-severe depressive symptoms in the past 2 weeks (defined as having PHQ-9 score ≥10) and substance use disorder in past year (defined as having a DAST-10 score ≥3).

### Patient and public involvement statement

Several patient and public organisations were involved in participant engagement, management and dissemination of the multicentre HIV-incidence study during 2019–2022.^11^ The original research protocol was developed after consultation with a number of community-based organisations representing key populations in Nairobi, Malindi, Mombasa and Kisumu, including the Sex Workers Outreach Programme, HAPA-Kenya and Malindi Desire Initiative. When funds were obtained for CT/NG testing, we consulted representatives of these same organisations about a protocol amendment to add testing for CT/NG infections in cohort participants. At study closure, we presented findings of this research directly to participants at public meetings at each of the above-named organisations.

### Statistical analysis

Baseline characteristics for those with and without CT/NG infection at any site were compared using Pearson’s χ^2^ or Fisher’s exact tests as appropriate. Prevalence of CT/NG combined, CT and NG at any site and at specific anatomic sites at baseline was similarly compared across groups. Associations of gender identity (ie, MSM vs TGW) and other potential correlates with prevalent CT/NG infection at any site at baseline were examined using Poisson regression with clustering by study site and robust error variances to generate prevalence ratios (PR) with 95% CIs.

Incidence rates (IRs) were estimated separately for MSM and TGW. In addition, IRs of CT, NG and CT/NG infection overall were calculated, and stratified by baseline infection and anatomical site. Poisson regression with clustering by study site and robust error variances was used to explore associations of group (ie, MSM vs TGW) and other potential correlates with incident CT/NG infection, generating IR ratios (IRRs) and their 95% CI.

Conceptualised confounders of the association between gender identity and prevalent or incident CT/NG included age, condom use for RAI/IAI, condom use during last sex, RAI, IAI, sexual partner type for the last male and last female partner reported, number of male and female partners, receiving payment for sex, paying for sex and PrEP use. These potential confounders were assessed for multicollinearity using variance inflation factor (VIF), with only one variable included in the model when a pair of potential confounders had a VIF of 5 or more. After creating an initial model with all potential confounders included, we selected the most parsimonious model (ie, the model with the lowest Bayesian Information Criterion) for each outcome.^14^ We determined a priori to include the variable ‘receiving payment for sex’ in our models, rather than sexual partner type for last male and for last female partner, due to the conceptual overlap in these three variables. To assess the correlates of incident CT/NG infection separately for TGW and MSM, we also fitted the final multivariable model stratified by gender identity. Data were cleaned and analysed using Stata V.18.0 (StataCorp LLC, College Station, Texas, USA).

## Results

### Study population

A total of 650 participants underwent CT/NG testing at baseline, and 112 (17.2%) participants had a CT/NG infection at any site. There were 570 (87.7%) MSM and 80 (12.3%) TGW. The prevalence of CT/NG infection at baseline was higher among participants at Mtwapa site, TGW and those that reported their last male sexual partner as transactional and reported receiving payment for sex (table 1).

**Table 1:** Characteristics of the study population, overall and by chlamydia and/or gonorrhoea status

Characteristics	Overall (n=650)		Infection with CT or NG at any anatomic site				P value
			Negative (n=538)		Positive (n=112)		
	N (column %)		N (row %)		N (row %)		
Study site							**0.038**
Kisumu	245	(37.7)	203	(82.9)	42	(17.1)	
Nairobi	214	(32.9)	180	(84.1)	34	(15.9)	
Mtwapa	94	(14.5)	69	(73.4)	25	(26.6)	
Malindi	97	(14.9)	86	(88.7)	11	(11.3)	
Gender identity							**0.009**
MSM	570	(87.7)	480	(84.2)	90	(15.8)	
TGW	80	(12.3)	58	(72.5)	22	(27.5)	
Age (years)							0.192
18–24	306	(47.1)	247	(80.7)	59	(19.3)	
≥25	344	(52.9)	291	(84.6)	53	(15.4)	
Ever married to a female							0.521
No	569	(87.5)	473	(83.1)	96	(16.9)	
Yes	81	(12.5)	65	(80.2)	16	(19.8)	
Educational attainment							0.279
Primary	110	(16.9)	86	(78.2)	24	(21.8)	
Secondary	381	(58.6)	322	(84.5)	59	(15.5)	
Higher/tertiary/other	159	(24.5)	130	(81.8)	29	(18.2)	
Employment							0.207
Unemployed	362	(55.7)	291	(80.4)	71	(19.6)	
Employed	131	(20.2)	109	(83.2)	22	(16.8)	
Self-employed	155	(23.8)	136	(87.7)	19	(12.3)	
Missing response	2	(0.3)	2	(100.0)	0	(0.0)	
Gender of last sexual partner							0.444
Male	532	(81.8)	436	(82.0)	96	(18.0)	
Female	116	(17.8)	100	(86.2)	16	(13.8)	
Missing response	2	(0.3)	2	(100.0)	0	(0.0)	
Last male sexual partner category							**0.001**
Regular	396	(60.9)	341	(86.1)	55	(13.9)	
Casual	121	(18.6)	102	(84.3)	19	(15.7)	
Paying/paid	126	(19.4)	89	(70.6)	37	(29.4)	
Missing response	7	(1.1)	6	(85.7)	1	(14.3)	
Last female sexual partner category							0.202
Regular	322	(49.5)	277	(86.0)	45	(14.0)	
Casual	141	(21.7)	114	(80.9)	27	(19.1)	
Paying/paid	116	(17.8)	93	(80.2)	23	(19.8)	
Missing response	71	(10.9)	54	(76.1)	17	(23.9)	
Total number of male partners, past 3 months							0.149
None	62	(9.5)	58	(93.5)	4	(6.5)	
One	134	(20.6)	112	(83.6)	22	(16.4)	
Two or more	446	(68.6)	362	(81.2)	84	(18.8)	
Missing response	8	(1.2)	6	(75.0)	2	(25.0)	
Total number of female partners, past 3 months							0.715
None	207	(31.8)	168	(81.2)	39	(18.8)	
One	160	(24.6)	138	(86.3)	22	(13.8)	
Two or more	256	(39.4)	210	(82.0)	46	(18.0)	
Missing response	27	(4.2)	22	(81.5)	5	(18.5)	
Receptive anal intercourse, past 3 months							0.301
No	348	(53.5)	293	(84.2)	55	(15.8)	
Yes	302	(46.5)	245	(81.1)	57	(18.9)	
Condom use for receptive anal intercourse, past 3 months							0.126
No	57	(8.8)	46	(80.7)	11	(19.3)	
Yes	244	(37.5)	199	(81.6)	45	(18.4)	
No receptive anal intercourse	348	(53.5)	293	(84.2)	55	(15.8)	
Missing response	1	(0.2)	0	(0.0)	1	(100.0)	
Insertive anal intercourse, past 3 months							0.868
No	147	(22.6)	121	(82.3)	26	(17.7)	
Yes	503	(77.4)	417	(82.9)	86	(17.1)	
Condom use for insertive anal intercourse, past 3 months							0.986
No	82	(12.6)	68	(82.9)	14	(17.1)	
Yes	421	(64.8)	349	(82.9)	72	(17.1)	
No insertive anal intercourse	147	(22.6)	121	(82.3)	26	(17.7)	
Condom use during last sex							0.567
No	148	(22.8)	119	(80.4)	29	(19.6)	
Yes	500	(76.9)	417	(83.4)	83	(16.6)	
Missing response	2	(0.3)	2	(100.0)	0	(0.0)	
Receiving payment for sex, past 3 months							**0.028**
No	300	(46.2)	260	(86.7)	40	(13.3)	
Yes	348	(53.5)	277	(79.6)	71	(20.4)	
Missing response	2	(0.3)	1	(50.0)	1	(50.0)	
Paid for sex, past 3 months							0.826
No	411	(63.2)	342	(83.2)	69	(16.8)	
Yes	238	(36.6)	195	(81.9)	43	(18.1)	
Missing response	1	(0.2)	1	(100.0)	0	(0.0)	
Self-reported daily oral PrEP use							0.352
No PrEP use	496	(76.3)	416	(83.9)	80	(16.1)	
Taking PrEP	153	(23.5)	121	(79.1)	32	(20.9)	
Missing response	1	(0.2)	1	(100.0)	0	(0.0)	
Disordered alcohol use (AUDIT), past year							0.524
Low (0–7)	446	(68.6)	372	(83.4)	74	(16.6)	
Hazardous (8–40)	204	(31.4)	166	(81.4)	38	(18.6)	
Problematic substance use (DAST-10), past year							0.083
No (0–2)	440	(67.7)	372	(84.5)	68	(15.5)	
Yes (≥3)	210	(32.3)	166	(79.0)	44	(21.0)	
Depressive symptoms (PHQ-9), past 2 weeks							0.170
Minimal to mild (0–9)	550	(84.6)	460	(83.6)	90	(16.4)	
Moderate to severe (10–27)	100	(15.4)	78	(78.0)	22	(22.0)	
Urogenital symptoms, past 3 months							0.842
No	580	(89.2)	481	(82.9)	99	(17.1)	
Yes	69	(10.6)	56	(81.2)	13	(18.8)	
Missing response	1	(0.2)	1	(100.0)	0	(0.0)	
Rectal symptoms, past 3 months							0.867
No	612	(94.2)	507	(82.8)	105	(17.2)	
Yes	37	(5.7)	30	(81.1)	7	(18.9)	
Missing response	1	(0.2)	1	(100.0)	0	(0.0)	
Oropharyngeal symptoms, past 3 months							0.561
No	616	(94.8)	512	(83.1)	104	(16.9)	
Yes	23	(3.5)	18	(78.3)	5	(21.7)	
Missing response	11	(1.7)	8	(72.7)	3	(27.3)	

Percentages are rounded to one decimal place. Due to rounding, total percentages may not sum to exactly 100%.

Bold P values < 0.05

AUDIT, Alcohol Use Disorder Identification Test; CT, *Chlamydia trachomatis*; DAST-10, Drug Abuse Screening Test 10; MSM, men who have sex with men; NG, *Neisseria gonorrhoeae*; PHQ-9, Patient Health Questionnaire 9; PrEP, pre-exposure prophylaxis; TGW, transgender women.

### CT/NG prevalence by group

Overall CT/NG infection prevalence was 15.8%, 95% CI (12.9% to 19.0%) and 27.5%, 95% CI (18.1% to 38.6%) in MSM and TGW, respectively (p=0.009). Compared with MSM, TGW had a higher prevalence of oropharyngeal CT/NG infection (15.2% vs 1.0%, p<0.001), NG infection at any site (8.8% vs 2.5%, p=0.002), and oropharyngeal NG infection (12.1% vs 1.0%, p=0.001) (data in online supplemental table S1).

### Factors associated with prevalent CT/NG infection

Table 2 presents an analysis of factors associated with prevalent CT/NG infection at any site of infection. TGW had a higher prevalence of CT/NG infection in bivariable analysis, compared with MSM (PR 1.77, 95% CI 1.10 to 2.84). This difference was attenuated in adjusted analyses (PR 1.61, 95% CI 0.99 to 2.62). Factors other than gender identity that were associated with prevalent CT/NG infection in bivariable analysis included last male sexual partner (p*=*0.005), and receiving payment for sex in the past 3 months (p=0.057). In the adjusted multivariable analysis, no factors were significantly associated with prevalent CT/NG infection.

**Table 2:** Risk factors associated with prevalent CT and/or NG infection at any site among 650 HIV-negative cisgender men who have sex with men and transgender women

Characteristics	Bivariable analysis		Multivariable analysis*, n=650	
	PR (95% CI)	P value	aPR (95% CI)	P value
Gender identity		**0.018**		0.056
MSM	Reference		Reference	
TWG	**1.77 (1.10 to 2.84**)		1.61 (0.99 to 2.62)	
Age (years)		0.197		0.178
18–24	Reference		Reference	
≥25	0.78 (0.53 to 1.14)		0.77 (0.52 to 1.13)	
Ever married to a female		0.559		
No	Reference			
Yes	1.17 (0.69 to 1.99)			
Educational attainment		0.355		
Primary	Reference			
Secondary	0.71 (0.44 to 1.14)			
Higher/tertiary/other	0.83 (0.48 to 1.45)			
Employment		0.334		
Unemployed	Reference			
Employed	0.85 (0.52 to 1.37)			
Self-employed	0.62 (0.37 to 1.04)			
Missing response	†			
Gender of last sexual partner		0.601		
Male	Reference			
Female	0.76 (0.47 to 1.29)			
Missing response	†			
Last male sexual partner category		**0.005**		
Regular	Reference			
Casual	1.13 (0.67 to 1.90)			
Paying/paid	**2.11 (1.39 to 3.21**)			
Missing response	1.03 (0.14 to 7.43)			
Last female sexual partner category		0.201		
Regular	Reference			
Casual	1.37 (0.84 to 2.22)			
Paying/paid	1.42 (0.85 to 2.35)			
Missing response	1.74 (0.99 to 3.04)			
Total number of male partners, past 3 months		0.190		0.431
None	0.39 (0.14 to 1.14)		0.42 (0.14 to 1.23)	
One	Reference		Reference	
Two or more	1.15 (0.72 to 1.83)		0.95 (0.57 to 1.59)	
Missing response	1.52 (0.36 to 6.48)		1.29 (0.26 to 6.34)	
Total number of female partners, past 3 months		0.666		
None	1.38 (0.81 to 2.32)			
One	Reference			
Two or more	1.31 (0.79 to 2.19)			
Missing response	1.35 (0.51 to 3.57)			
Receptive anal intercourse, past 3 months		0.348		0.913
No	Reference		Reference	
Yes	1.19 (0.82 to 1.73)		0.96 (0.65 to 1.43)	
Condom use for receptive anal intercourse, past 3 months		0.279		
No	1.03 (0.53 to 2.02)			
Yes	Reference			
No receptive anal intercourse	0.86 (0.58 to 1.29)			
Missing response	5.70 (0.72 to 45.48)			
Insertive anal intercourse, past 3 months		0.880		
No	Reference			
Yes	0.97 (0.62 to 1.50)			
Condom use for insertive anal intercourse, past 3 months		0.987		
No	0.98 (0.54 to 1.77)			
Yes	Reference			
No insertive anal intercourse	1.03 (0.66 to 1.62)			
Condom use during last sex		0.768		0.729
No	Reference		Reference	
Yes	0.85 (0.55 to 1.32)		0.86 (0.55 to 1.34)	
Missing response	†		†	
Receiving payment for sex, past 3 months		0.057		0.169
No	Reference		Reference	
Yes	**1.53 (1.04 to 2.26**)		1.38 (0.90 to 2.12)	
Missing response	3.84 (0.52 to 28.37)		4.03 (0.45 to 36.37)	
Paid for sex, past 3 months		0.927		
No	Reference			
Yes	1.08 (0.74 to 1.58)			
Missing response	†			
Self-reported daily oral PrEP use		0.462		0.511
No PrEP use	Reference		Reference	
Taking PrEP	1.30 (0.86 to 1.96)		1.29 (0.84 to 1.98)	
Missing response	†		†	
Disordered alcohol use (AUDIT), past year		0.580		
Low (0–7)	Reference			
Hazardous (8–40)	1.12 (0.75 to 1.66)			
Problematic substance use (DAST-10), past year		0.113		
No (0–2)	Reference			
Yes (≥3)	1.36 (0.93 to 1.99)			
Depressive symptoms (PHQ-9), past 2 weeks		0.213		
Minimal to mild (0–9)	Reference			
Moderate to severe (10–27)	1.34 (0.84 to 2.14)			
Any urethral, anal or oropharyngeal symptoms, past 3 months		0.407		
No	Reference			
Yes	1.23 (0.76 to 1.99)			

Bold values indicate variables whose 95% CI of the PR did not cross 1.

* Model of conceptualised confounders of the association between gender identity and prevalent or incident CT/NG including payment for sex, a priori rather than sexual partner type for last male and for last female partner, due to the conceptual overlap in these three variables.

† No prevalent infection among participants who had a ‘missing response’. PR (95% CI)=<0.00 (0.00 to not determined) or >100.00 (0.00 to not determined).

aPR, adjusted prevalence ratio; AUDIT, Alcohol Use Disorder Identification Test; CT, *Chlamydia trachomatis*; DAST-10, Drug Abuse Screening Test 10; MSM, men who have sex with men; NG, *Neisseria gonorrhoeae*; PHQ-9, Patient Health Questionnaire 9; PR, prevalence ratio; PrEP, pre-exposure prophylaxis; TGW, transgender women.

### CT/NG incidence overall and by group

Table 3 presents IRs for CT/NG at any site, by site of infection, and by gender identity, excluding the seven participants who did not have treatment for their baseline infection. A total of 528 participants contributed 268.3 person-years (PY) of follow-up time, and 72 participants had incident CT/NG infection, for an IR of 26.8 (95% CI 21.3 to 33.8) per 100 PY. The IR per 100 PY for incident CT/NG infection at any site was not statistically significantly different between MSM and TGW (p=0.784).

**Table 3:** Incidence of urogenital, rectal and oropharyngeal CT and/or NG infections at any site among 528 HIV-negative cisgender men who have sex with men and transgender women

Site of infection	Overall		MSM		TGW		
	Cases/100 PY	Incidence rate/100 PY (95% CI)	Cases/100 PY	Incidence rate/100 PY (95% CI)	Cases/100 PY	Incidence rate/100 PY (95% CI)	P value
CT/NG infection*	72/268.3	26.8 (21.3 to 33.8)	64/235.7	27.2 (21.3 to 34.7)	8/32.6	24.5 (12.3 to 49.0)	0.784
Any CT/NG infection at baseline							
Negative	44/221.9	19.8 (14.8 to 26.6)	39/197.0	19.8 (14.5 to 27.1)	5/24.9	20.1 (8.3 to 48.2)	
Positive	28/46.4	60.4 (41.7 to 87.4)	25/38.7	64.6 (43.7 to 95.6)	3/7.7	38.9 (12.5 to 120.6)	
Urogenital	46/267.9	17.2 (12.9 to 22.9)	45/235.3	19.1 (14.3 to 25.6)	1/32.6	3.1 (0.4 to 21.7)	0.070
Rectal	39/264.0	14.8 (10.8 to 20.2)	32/231.4	13.8 (9.8 to 19.6)	7/32.6	21.4 (10.2 to 45.0)	0.293
Oropharyngeal†	1/85.3	1.2 (0.2 to 8.3)	0/73.4	‡	1/11.9	8.4 (1.2 to 59.4)	–
CT infection	65/268.3	24.2 (19.0 to 30.9)	59/235.7	25.0 (19.4 to 32.3)	6/32.6	18.4 (8.3 to 40.9)	0.471
Any CT infection at baseline							
Negative	37/225.7	16.4 (11.9 to 22.6)	34/198.5	17.1 (12.2 to 24.0)	3/27.2	11.0 (3.6 to 34.2)	
Positive	28/42.6	65.7 (45.4 to 95.2)	25/37.2	67.3 (45.5 to 99.6)	3/5.4	55.3 (17.8 to 171.3)	
Urogenital	45/267.9	16.8 (12.5 to 22.5)	44/235.3	18.7 (13.9 to 25.1)	1/32.6	3.1 (0.4 to 21.7)	0.074
Rectal	32/264.0	12.1 (8.6 to 17.1)	27/231.4	11.7 (8.0 to 17.0)	5/32.6	15.3 (6.4 to 36.8)	0.576
Oropharyngeal†	0/84.2	‡	0/72.5	‡	0/11.7	‡	–
NG infection	10/268.3	3.7 (2.0 to 6.9)	7/235.7	3.0 (1.4 to 6.2)	3/32.6	9.2 (3.0 to 28.5)	0.102
Any NG infection at baseline							
Negative	10/260.2	3.7 (2.0 to 6.9)	7/230.2	3.0 (1.4 to 6.4)	3/29.9	10.0 (3.2 to 31.1)	
Positive	0/8.1	‡	0/5.4	‡	0/2.7	‡	–
Urogenital	2/267.9	0.7 (0.2 to 3.0)	2/235.3	0.9 (0.2 to 3.4)	0/32.6	‡	–
Rectal	10/264.0	3.8 (2.0 to 7.0)	7/231.4	3.0 (1.4 to 6.3)	3/32.6	9.2 (3.0 to 28.5)	0.107
Oropharyngeal†	1/85.1	1.2 (0.2 to 8.3)	0/73.2	‡	1/11.9	8.4 (1.2 to 59.4)	

* Participants (n=7) who tested positive CT/NG at baseline with no record of treatment were excluded from incidence analysis.

† Oropharyngeal infections in random (1/3) sample of participants.

‡ No incident infection among participants. IR (95% CI)=0.0 (not determined).

CT, *Chlamydia trachomatis*; IR, incidence rate; MSM, men who have sex with men; NG, *Neisseria gonorrhoeae*; PY, person-years; TGW, transgender women.

Overall, the IR per 100 PY of CT infection at any site was 24.2 (95%CI 19.0 to 30.9), and of NG infection at any site was 3.7 (95%CI 2.0 to 6.9). There was no statistically significant difference in CT incidence between MSM and TGW, although the IR per 100 PY for urethral CT infection was higher among MSM (18.7, 95% CI 13.9 to 25.1) compared with TGW (3.1, 95% CI 0.4 to 21.7), with borderline significance (p=0.074).

The IR per 100 PY for NG infection at any site was lower among MSM (3.0, 95% CI 1.4 to 6.2) compared with TGW (9.2, 95% CI 3.0 to 28.5, p=0.102) but not statistically significantly different. While no urogenital incident infections occurred in TGW, TGW had an approximately threefold higher NG IR per 100 PY relative to MSM for the combined rectal and oropharyngeal sites. The IRR for TGW compared with MSM did not differ in unadjusted (IRR=0.90; 95% CI 0.43 to 1.88) or adjusted IRR (aIRR=0.74; 95% CI 0.34 to 1.61) analyses (table 4).

**Table 4:** Risk factors associated with incident CT and/or NG infection at any site among 528 HIV-negative cisgender men who have sex with men and transgender women

Characteristics	Cases, n=72	Bivariable analysis		Multivariable analysis*, n=528	
	N/100 PY (rate)	IRR (95% CI)	P value	aIRR (95% CI)	P value
Gender identity			0.784		0.451
MSM	64/235.7 (27.2)	Reference		Reference	
TGW	8/32.6 (24.5)	0.90 (0.43 to 1.88)		0.74 (0.34 to 1.61)	
Age (years)			0.907		0.564
18–24	30/110.0 (27.3)	Reference		Reference	
≥25	42/158.3 (26.5)	0.97 (0.61 to 1.55)		0.87 (0.54 to 1.40)	
Ever married to a female			0.678		
No	62/235.3 (26.3)	Reference			
Yes	10/33.0 (30.3)	1.15 (0.59 to 2.25)			
Educational attainment			0.898		
Primary	14/47.3 (29.6)	Reference			
Secondary	42/157.1 (26.7)	0.90 (0.49 to 1.65)			
Higher/tertiary/other	16/64.0 (25.0)	0.84 (0.41 to 1.73)			
Employment			0.696		
Unemployed	39/149.7 (24.0)	Reference			
Employed	19/56.2 (33.8)	1.40 (0.81 to 2.45)			
Self-employed	17/61.9 (27.5)	1.14 (0.64 to 2.03)			
Missing response	0/0.5 (0.0)	†			
Gender of last sexual partner			0.447		
Male	51/206.3 (24.7)	Reference			
Female	21/61.1 (34.4)	1.39 (0.84 to 2.31)			
Missing response	0/0.9 (0.0)	†			
Last male sexual partner category			0.281		
Regular	46/170.3 (27.0)	Reference			
Casual	9/52.1 (17.3)	0.64 (0.31 to 1.31)			
Paying/paid	17/43.5 (39.1)	1.42 (0.81 to 2.47)			
Missing response	0/1.5 (0.0)	†			
Last female sexual partner category			0.371		
Regular	43/139.2 (30.9)	Reference			
Casual	16/59.7 (26.8)	0.87 (0.49 to 1.54)			
Paying/paid	9/36.5 (24.6)	0.76 (0.37 to 1.56)			
Missing response	4/31.2 (12.8)	0.42 (0.15 to 1.16)			
Total number of male partners, past 3 months			0.340		0.352
None	11/25.1 (43.8)	1.44 (0.68 to 3.05)		1.30 (0.60 to 2.84)	
One	18/59.2 (30.4)	Reference		Reference	
Two or more	43/179.3 (24.0)	0.79 (0.46 to 1.37)		0.69 (0.38 to 1.25)	
Missing response	0/4.7 (0.0)	†		†	
Total number of female partners, past 3 months			0.906		
None	24/81.6 (29.4)	1.26 (0.68 to 2.35)			
One	17/72.9 (23.3)	Reference			
Two or more	28/101.9 (27.5)	1.18 (0.64 to 2.15)			
Missing response	3/11.9 (25.0)	1.08 (0.32 to 3.68)			
Receptive anal intercourse, past 3 months			0.459		0.383
No	32/138.3 (23.1)	Reference		Reference	
Yes	40/128.6 (31.1)	1.34 (0.84 to 2.14)		1.43 (0.86 to 2.36)	
Missing response	0/1.4 (0.0)	†		†	
Condom use for receptive anal intercourse, past 3 months			0.351		
No	10/22.5 (44.5)	1.58 (0.77 to 3.23)			
Yes	30/106.6 (28.1)	Reference			
No RAI	32/138.3 (23.1)	0.82 (0.50 to 1.35)			
Missing response	0/1.4 (0.0)	†			
Insertive anal intercourse, past 3 months			0.775		
No	19/61.1 (31.1)	Reference			
Yes	53/206.3 (25.7)	0.83 (0.49 to 1.40)			
Missing response	0/0.9 (0.0)	†			
Condom use for insertive anal intercourse, past 3 months			0.915		
No	9/36.1 (25.0)	0.97 (0.47 to 1.98)			
Yes	44/170.2 (25.8)	Reference			
No IAI	19/61.1 (31.1)	1.20 (0.70 to 2.06)			
Missing response	0/0.9 (0.0)	†			
Condom use during last sex			0.380		0.304
No	21/59.6 (35.2)	Reference		Reference	
Yes	51/207.7 (24.5)	0.70 (0.42 to 1.16)		0.66 (0.40 to 1.12)	
Missing response	0/0.9 (0.0)	†		†	
Receiving payment for sex, past 3 months			0.192		0.248
No	41/123.3 (33.3)	Reference		Reference	
Yes	31/143.6 (21.6)	0.65 (0.41 to 1.03)		0.64 (0.38 to 1.08)	
Missing response	0/1.4 (0.0)	†		†	
Paid for sex, past 3 months			0.964		
No	48/173.6 (27.7)	Reference			
Yes	24/92.9 (25.8)	0.93 (0.57 to 1.53)			
Missing response	0/1.9 (0.0)	†			
Self-reported daily oral PrEP use			0.162		**0.039**
Off-PrEP	47/201.0 (23.4)	Reference		Reference	
On-PrEP	24/65.4 (36.7)	1.57 (0.96 to 2.56)		**1.75 (1.05 to 2.93**)	
Missing response	1/1.9 (52.3)	2.24 (0.31 to 16.22)		5.23 (0.68 to 40.11)	
Disordered alcohol use (AUDIT), past year			0.916		
Low (0–7)	49/175.7 (27.9)	Reference			
Hazardous (8–40)	23/91.7 (25.1)	0.90 (0.55 to 1.48)			
Missing response	0/0.9 (0.0)	†			
Problematic substance use (DAST-10), past year			0.704		
No (0–2)	50/180.7 (27.7)	Reference			
Yes (≥3)	22/87.6 (25.1)	0.91 (0.55 to 1.50)			
Depressive symptoms (PHQ-9), past 2 weeks			0.683		
Minimal to mild (0–9)	62/235.3 (26.4)	Reference			
Moderate to severe (10–27)	10/33.0 (30.3)	1.15 (0.59 to 2.24)			
CT/NG infection at any site at baseline‡			**<0.001**		**<0.001**
No	44/221.9 (19.8)	Reference		Reference	
Yes	28/46.4 (60.4)	**3.04 (1.90 to 4.89**)		**3.14 (1.94 to 5.07**)	
Any urethral, anal or oropharyngeal symptoms, past 3 months			0.700		
No	63/230.5 (27.3)	Reference			
Yes	9/37.8 (23.8)	0.87 (0.43 to 1.75)			

Bold values indicate variables whose 95% CI of the IRR did not cross 1.

* Model of conceptualised confounders of the association between gender identity and prevalent or incident CT/NG including payment for sex, a priori rather than sexual partner type for last male and for last female partner, due to the conceptual overlap in these three variables.

† No incident infection among participants who had a ‘missing response’. IRR (95% CI)=<0.00 (0.00 to not determined) or >100.00 (0.00 to not determined).

‡ Participants (n=7) who tested positive CT/NG at baseline with no record of treatment were excluded from incidence analysis.

aIRR, adjusted incidence rate ratio; AUDIT, Alcohol Use Disorder Test; CT, *Chlamydia trachomatis*; DAST-10, Drug Abuse Screening Test 10; IAI, insertive anal intercourse; IRR, incidence rate ratio; MSM, men who have sex with men; NG, *Neisseria gonorrhoeae*; PHQ-9, Patient Health Questionnaire 9; PrEP, pre-exposure prophylaxis; PY, person-years; RAI, receptive anal intercourse; TGW, transgender women.

For participants who had a baseline CT/NG infection with documented treatment, the IR was 60.4 (95% CI 41.7 to 87.4) per 100 PY, while for participants who were CT/NG negative at baseline, the IR was 19.8 (95% CI 14.8 to 26.6) per 100 PY. This difference was driven by repeat CT infections, as no new NG infections occurred in participants with an NG infection at baseline (table 3).

### Risk factors for any incident CT/NG infection

Table 4 presents an analysis of factors associated with incident CT/NG infection at any site. In multivariable analysis, participants with CT/NG infection at baseline were 3.1 times more likely to acquire CT/NG infection during follow-up (aIRR 3.14, 95% CI 1.94 to 5.07) despite treatment, compared with participants without CT/NG infection at baseline. Participants self-reporting PrEP use had over 75% increased risk of incident CT/NG infection (aIRR 1.75, 95% CI 1.05 to 2.93) as compared with those self-reporting not using PrEP.

### Sensitivity analysis for risk factors for any incident CT/NG infection by group

In multivariable analysis of 465 MSM, only CT/NG infection at baseline was associated with incident CT/NG infection during follow-up (aIRR 3.27, 95% CI 1.95 to 5.49), compared with participants without CT/NG infection at baseline. Among 63 TGW, no factors were associated with CT/NG infection during follow-up (data in online supplemental table S2).

Three participants (all MSM) acquired HIV for an estimated HIV incidence of 1.1 (95% CI 0.4 to 3.9) per 100 PY. Of the three participants who acquired HIV, one had a baseline CT/NG infection approximately 70 days prior to the estimated date of HIV acquisition. All three participants had negative CT/NG test results at their month six retesting.

## Discussion

We documented a high burden of CT/NG infection at rectal, urethral or oropharyngeal sites among 18–29 years old MSM and TGW participating at three research centres in Kenya, with one in seven MSM and more than one in four TGW having prevalent CT/NG infection at any site at baseline. MSM and TGW had comparable CT/NG incidence over follow-up (27–25% of MSM and TGW, respectively), with no significant difference in this outcome in unadjusted or adjusted analysis. Crude NG incidence was threefold higher in TGW compared with MSM, at 9.0 per 100 PY compared with 3.0 per 100 PY, although not statistically significantly different. The high NG incidence in TGW was comparable to a recent NG incidence estimate of 11.4 per 100 PY in young women 16–25 years eligible for PrEP in the HPTN (HIV Prevention Trials Network) 082 trial in South Africa and Zimbabwe.^15^ TGW also had higher crude rates than MSM of oropharyngeal NG infections.

While TGW had higher CT/NG prevalence by several measures at baseline and the higher NG incidence in TGW was pronounced, we did not find differences in CT/NG prevalence and incidence between TGW and MSM in this cohort, after adjustment for potential confounders of the relationship between gender identity and our primary outcomes. In an earlier study from the same cohort, we showed that TGW reported a higher frequency of behaviours associated with potential exposure to STI and HIV (eg, RAI, group sex, multiple male partners, multiple male transactional sex partners) than MSM and also had higher rates of moderate to severe depressive symptoms, but not of unhealthy alcohol or other substance use.^10^ More attention to the vulnerabilities and needs of TGW in Kenya and other settings in which gender-affirming care is not the norm is clearly needed.

We identified two predictors of incident CT/NG infection at any site in this population of MSM and TGW, including prevalent CT/NG at baseline and PrEP use. It is encouraging that approximately one in four individuals at high risk for STIs report using PrEP; among PrEP-using individuals, globally, STI rates have been substantial, leading to calls for better integration of STI and HIV services.^16^ That prior CT/NG infection was predictive of new CT/NG infections despite presumably effective treatment of the participant and the offer of EPT, suggesting treatment failure of the partner, failure to adhere to treatment, ongoing high rates of STI within networks and insufficient partner treatment. Although we did not track doxycycline adherence or the uptake of EPT receipt in our cohort, our future studies will attempt to capture this data.

While our data highlight an urgent unmet need for expanded STI diagnosis and prevention among MSM and TGW, options for CT/NG screening and treatment are limited due to resource constraints in settings such as Kenya. Most bacterial STIs are asymptomatic,^17^ and NAAT CT/NG testing is prohibitively expensive in resource-limited settings.^18^ Notably, there is debate in well-resourced settings about the risks and benefits of frequent screening for CT and NG in asymptomatic MSM and TGW, especially in view of emerging antimicrobial resistance of NG.^19^ To investigate the value of screening, a recent trial in Belgium randomised over 1000 MSM and TGW participants taking PrEP to either 3-monthly NAAT screening of three anatomical sites or to no CT/NG screening.^20^ This study showed that screening was associated with higher antibiotic consumption and had no effect on NG incidence, but failed to show that no screening was non-inferior to screening in this population.^20^

One potential alternative to NAAT-based testing strategies in resource-limited settings is periodic presumptive treatment (PPT) of CT/NG infections in MSM. In 2011, the WHO recommended PPT to eradicate asymptomatic CT and NG infections among MSM and TGW who report condomless RAI and either multiple sex partners or a sex partner with an STI in the past 6 months; no frequency of this intervention was recommended.^21^ A 2013 study among MSM from coastal Kenya meeting WHO criteria for PTT estimated the number needed to treat one CT/NG infection as four.^22^ More recently, doxycycline post-exposure prophylaxis (doxyPEP, ie, 200 mg oral doxycycline taken within 24–72 hours following unprotected intercourse) has been found to reduce incidence of a combined CT, NG and syphilis infection outcome among MSM and TGW in three randomised clinical trials conducted in France and the USA.^23^,^25^ Neither WHO-recommended PPT nor doxyPEP has been evaluated among MSM or TGW in sSA, where NG resistance to doxycycline is almost universal (97%).^26^ A doxyPEP trial among young women taking HIV PrEP in Kenya had no effect on CT, syphilis and NG incidence,^27^ likely caused by insufficient adherence to doxycycline and high levels of doxycycline resistance in NG.^27 28^ It should be noted that regular use of doxyPEP is relatively costly compared with infrequent syndromic treatment as needed, leads to increased antibiotic consumption and may induce resistance in non-STI pathogens.^29^ In Kenya, treatment of syphilis and syndromic STI is provided free of charge to patients, whereas doxyPEP is currently unavailable unless patients pay out of pocket.

Many MSM, some TGW and their male sexual partners often have female sexual partners, potentially posing a risk of bridging transmission from men to women and vice versa and impacting infertility and neonatal infection.^30^ STI prevention and treatment among MSM in sSA should include notification of both male and female partners with assistance from healthcare providers,^31 32^ along with education that HIV PrEP does not prevent STI.^16^ In addition, providers should be made aware that adolescent and young adults, including young MSM, are disproportionately affected by STIs and need regular screening.^2^

Our study had a number of strengths, including that participants were drawn from a multicentre study at three regions in Kenya, that the study team was experienced in MSM research and had a long history of engagement with this community, and that site-specific procedures included electronic verification of participants’ identity through iris scanning to prevent co-enrolment. Our study also had some limitations, including that CT/NG testing was not the original purpose of this HIV incidence cohort;^11^ that only a limited number of oropharyngeal samples were obtained and tested due to limited funding, making our oropharyngeal CT/NG prevalence and incidence estimates uncertain; and that no record was kept on partners’ receipt of EPT. Because the focus of our study was not on the effectiveness of the STI regimens we used, we did not collect data on doxycycline adherence or NG antimicrobial resistance, and did not conduct tests of cure. Based on a few HIV incidence infections, we were not able to evaluate relationships between CT/NG infections and HIV.

In conclusion, we documented a high prevalence and incidence of CT/NG infection across anatomic sites among young adult HIV-negative MSM and TGW in Kenya. CT/NG prevalence and NG incidence were higher among TGW, but did not differ after adjustment for sexual behaviours. Prevalent CT/NG infection at baseline and self-reported PrEP use were important determinants of incident CT/NG infection. New strategies need to be evaluated to reduce the burden of CT/NG infections in MSM and TGW in sSA.

## Supplementary material

10.1136/bmjopen-2025-098916 online supplemental file 1

## Data Availability

Data are available upon reasonable request.
