# Supplementary material for: Chlamydia and gonorrhoea infections in young Kenyan HIV-negative cisgender men who have sex with men and transgender women: a multicentre cohort study
Source: BMJ Open. 2025 Aug 1;15(7):e098916. doi: 10.1136/bmjopen-2025-098916 (PMC12314943; doi:10.1136/bmjopen-2025-098916)
Supplement: online supplemental file 1 [file bmjopen-15-7-s001.docx]

**Table S1. Characteristics of the study population, overall and by gender identity**

| **Characteristics** | | **Overall (n = 650)** | | **MSM (n = 570)** | | **TGW (n = 80)** | | ***P* value** |
| --- | --- | --- | --- | --- | --- | --- | --- | --- |
|  |  | **n (Column %)** | | **n (Column %)** | | **n (Column %)** | |  |
| Study site | |  |  |  |  |  |  | **<0.001** |
|  | Kisumu | 245 | (37.7) | 213 | (37.4) | 32 | (40.0) |  |
|  | Nairobi | 214 | (32.9) | 200 | (35.1) | 14 | (17.5) |  |
|  | Mtwapa | 94 | (14.5) | 83 | (14.6) | 11 | (13.8) |  |
|  | Malindi | 97 | (14.9) | 74 | (13.0) | 23 | (28.8) |  |
| Age (years) | |  |  |  |  |  |  | 0.202 |
|  | 18-24 | 306 | (47.1) | 263 | (46.1) | 43 | (53.8) |  |
|  | ≥25 | 344 | (52.9) | 307 | (53.9) | 37 | (46.3) |  |
| Ever married to a female | |  |  |  |  |  |  | 0.991 |
|  | No | 569 | (87.5) | 499 | (87.5) | 70 | (87.5) |  |
|  | Yes | 81 | (12.5) | 71 | (12.5) | 10 | (12.5) |  |
| Educational attainment | |  |  |  |  |  |  | **0.001** |
|  | Primary | 110 | (16.9) | 85 | (14.9) | 25 | (31.3) |  |
|  | Secondary | 381 | (58.6) | 342 | (60.0) | 39 | (48.8) |  |
|  | Higher/tertiary/other | 159 | (24.5) | 143 | (25.1) | 16 | (20.0) |  |
| Employment | |  |  |  |  |  |  | 0.685 |
|  | Unemployed | 362 | (55.7) | 317 | (55.6) | 45 | (56.3) |  |
|  | Employed | 131 | (20.2) | 118 | (20.7) | 13 | (16.3) |  |
|  | Self-employed | 155 | (23.8) | 133 | (23.3) | 22 | (27.5) |  |
|  | Missing response | 2 | (0.3) | 2 | (0.4) | 0 | (0.0) |  |
| Gender of last sexual partner | |  |  |  |  |  |  | 0.214 |
|  | Male | 532 | (81.8) | 465 | (81.6) | 67 | (83.8) |  |
|  | Female | 116 | (17.8) | 104 | (18.2) | 12 | (15.0) |  |
|  | Missing response | 2 | (0.3) | 1 | (0.2) | 1 | (1.3) |  |
| Last male sexual partner category | |  |  |  |  |  |  | **0.014** |
|  | Regular | 396 | (60.9) | 358 | (62.8) | 38 | (47.5) |  |
|  | Casual | 121 | (18.6) | 104 | (18.2) | 17 | (21.3) |  |
|  | Paying/paid | 126 | (19.4) | 101 | (17.7) | 25 | (31.3) |  |
|  | Missing response | 7 | (1.1) | 7 | (1.2) | 0 | (0.0) |  |
| Last female sexual partner category | |  |  |  |  |  |  | 367 |
|  | Regular | 322 | (49.5) | 289 | (50.7) | 33 | (41.3) |  |
|  | Casual | 141 | (21.7) | 122 | (21.4) | 19 | (23.8) |  |
|  | Paying/paid | 116 | (17.8) | 100 | (17.5) | 16 | (20.0) |  |
|  | Missing response | 70 | (10.8) | 58 | (10.2) | 12 | (15.0) |  |
| Total number of male partners, past 3 months | |  |  |  |  |  |  | 0.258 |
|  | None | 62 | (9.5) | 58 | (10.2) | 4 | (5.0) |  |
|  | One | 134 | (20.6) | 114 | (20.0) | 20 | (25.0) |  |
|  | Two or more | 446 | (68.6) | 392 | (68.8) | 54 | (67.5) |  |
|  | Missing response | 8 | (1.2) | 6 | (1.1) | 2 | (2.5) |  |
| Total number of female partners, past 3 months | |  |  |  |  |  |  | 0.530 |
|  | None | 207 | (31.8) | 185 | (32.5) | 22 | (27.5) |  |
|  | One | 160 | (24.6) | 143 | (25.1) | 17 | (21.3) |  |
|  | Two or more | 256 | (39.4) | 219 | (38.4) | 37 | (46.3) |  |
|  | Missing response | 27 | (4.2) | 23 | (4.0) | 4 | (5.0) |  |
| Receptive anal intercourse, past 3 months | |  |  |  |  |  |  | **0.010** |
|  | No | 348 | (53.5) | 316 | (55.4) | 32 | (40.0) |  |
|  | Yes | 302 | (46.5) | 254 | (44.6) | 48 | (60.0) |  |
| Condom use for receptive anal intercourse, past 3 months | |  |  |  |  |  |  | **<0.001** |
|  | No | 57 | (8.8) | 40 | (7.0) | 17 | (21.3) |  |
|  | Yes | 244 | (37.5) | 213 | (37.4) | 31 | (38.8) |  |
|  | No receptive anal intercourse | 348 | (53.5) | 316 | (55.4) | 32 | (40.0) |  |
|  | Missing response | 1 | (0.2) | 1 | (0.2) | 0 | (0.0) |  |
| Insertive anal intercourse, past 3 months | |  |  |  |  |  |  | 0.265 |
|  | No | 147 | (22.6) | 125 | (21.9) | 22 | (27.5) |  |
|  | Yes | 503 | (77.4) | 445 | (78.1) | 58 | (72.5) |  |
| Condom use for insertive anal intercourse, past 3 months | |  |  |  |  |  |  | 0.230 |
|  | No | 82 | (12.6) | 69 | (12.1) | 13 | (16.3) |  |
|  | Yes | 421 | (64.8) | 376 | (66.0) | 45 | (56.3) |  |
|  | No insertive anal intercourse | 147 | (22.6) | 125 | (21.9) | 22 | (27.5) |  |
| Condom use during last sex | |  |  |  |  |  |  | 0.138 |
|  | No | 148 | (22.8) | 123 | (21.6) | 25 | (31.3) |  |
|  | Yes | 500 | (76.9) | 445 | (78.1) | 55 | (68.8) |  |
|  | Missing response | 2 | (0.3) | 2 | (0.4) | 0 | (0.0) |  |
| Receiving payment for sex, past 3 months | |  |  |  |  |  |  | 0.419 |
|  | No | 300 | (46.2) | 268 | (47.0) | 32 | (40.0) |  |
|  | Yes | 348 | (53.5) | 300 | (52.6) | 48 | (60.0) |  |
|  | Missing response | 2 | (0.3) | 2 | (0.4) | 0 | (0.0) |  |
| Paid for sex, past 3 months | |  |  |  |  |  |  | 0.238 |
|  | No | 411 | (63.2) | 367 | (64.4) | 44 | (55.0) |  |
|  | Yes | 238 | (36.6) | 202 | (35.4) | 36 | (45.0) |  |
|  | Missing response | 1 | (0.2) | 1 | (0.2) | 0 | (0.0) |  |
| Self-reported daily oral PrEP use | |  |  |  |  |  |  | 0.327 |
|  | No PrEP use | 496 | (76.3) | 440 | (77.2) | 56 | (70.0) |  |
|  | Taking PrEP | 153 | (23.5) | 129 | (22.6) | 24 | (30.0) |  |
|  | Missing response | 1 | (0.2) | 1 | (0.2) | 0 | (0.0) |  |
| Disordered alcohol use (AUDIT), past year | |  |  |  |  |  |  | 0.116 |
|  | Low (0-7) | 446 | (68.6) | 385 | (67.5) | 61 | (76.3) |  |
|  | Hazardous (8-40) | 204 | (31.4) | 185 | (32.5) | 19 | (23.8) |  |
| Problematic substance use (DAST-10), past year | |  |  |  |  |  |  | **0.037** |
|  | No (0-2) | 440 | (67.7) | 394 | (69.1) | 46 | (57.5) |  |
|  | Yes (≥3) | 210 | (32.3) | 176 | (30.9) | 34 | (42.5) |  |
| Depressive symptoms (PHQ-9), past 2 weeks | |  |  |  |  |  |  | 0.222 |
|  | Minimal to mild (0-9) | 550 | (84.6) | 486 | (85.3) | 64 | (80.0) |  |
|  | Moderate to severe (10-27) | 100 | (15.4) | 84 | (14.7) | 16 | (20.0) |  |
| Combined CT/NG infection^1^ | | 112 | (17.2) | 90 | (15.8) | 22 | (27.5) | **0.009** |
|  | Urogenital | 85 | (13.1) | 71 | (12.5) | 14 | (17.5) | 0.402 |
|  | Rectal | 44 | (6.8) | 36 | (6.3) | 8 | (10.0) | 0.211 |
|  | Oropharyngeal | 7 | (3.0) | 2 | (1.0) | 5 | (15.2) | **<0.001** |
| CT infection^1^ | | 100 | (15.4) | 84 | (14.7) | 16 | (20.0) | 0.222 |
|  | Urogenital | 79 | (12.2) | 66 | (11.6) | 13 | (16.3) | 0.430 |
|  | Rectal | 38 | (5.8) | 32 | (5.6) | 6 | (7.5) | 0.324 |
|  | Oropharyngeal | 3 | (1.3) | 2 | (1.0) | 1 | (3.0) | 0.387 |
| NG infection^1^ | | 21 | (3.2) | 14 | (2.5) | 7 | (8.8) | **0.002** |
|  | Urogenital | 10 | (1.5) | 9 | (1.6) | 1 | (1.3) | 0.833 |
|  | Rectal | 10 | (1.5) | 7 | (1.2) | 3 | (3.8) | 0.088 |
|  | Oropharyngeal | 6 | (2.6) | 2 | (1.0) | 4 | (12.1) | **0.001** |
| Urogenital symptoms, past 3 months | |  |  |  |  |  |  | 0.584 |
|  | No | 580 | (89.2) | 511 | (89.6) | 69 | (86.3) |  |
|  | Yes | 69 | (10.6) | 58 | (10.2) | 11 | (13.8) |  |
|  | Missing response | 1 | (0.4) | 1 | (0.2) | 0 | (0.0) |  |
| Rectal symptoms, past 3 months | |  |  |  |  |  |  | 0.423 |
|  | No | 612 | (94.2) | 539 | (94.6) | 73 | (91.3) |  |
|  | Yes | 37 | (5.7) | 30 | (5.3) | 7 | (8.8) |  |
|  | Missing response | 1 | (0.4) | 1 | (0.2) | 0 | (0.0) |  |
| Oropharyngeal symptoms, past 3 months | | 23 | (3.5) | 17 | (3.0) | 6 | (7.5) | 0.099 |
|  | No | 616 | (94.8) | 544 | (95.4) | 72 | (90.0) |  |
|  | Yes | 23 | (3.5) | 17 | (3.0) | 6 | (7.5) |  |
|  | Missing response | 11 | (4.7) | 9 | (1.6) | 2 | (2.5) |  |

AUDIT=Alcohol Use Disorder Identification, CT=Chlamydia trachomatis, DAST-10=Drug Abuse Screening Test 10, IQR=Interquartile range, MSM= Men who have sex with men, NG=Neisseria gonorrhoeae, PHQ-9=Patient Health Questionnaire 9, PrEP=Pre-exposure prophylaxis, TGW= Transgender women.

^1^2 urethral swabs were missing and 648 had results available, 12 rectal swabs were missing and 638 had results available. Among 235 participants selected for testing, 2 NG oropharyngeal swabs were missing and 233 had results available, 3 CT oropharyngeal swabs were missing and 232 had results available. Oropharyngeal infections swabs were tested in a random (1/3) sample of participants.

Percentages are rounded to one decimal place. Due to rounding, total percentages may not sum to exactly 100%.

**Table S2. Stratified analysis of risk factors associated with incident CT and/or NG infection at any site by gender identity**

| **Characteristics** | | **MSM final multivariable analysis^2^, n = 465** | |  | **TGW final multivariable analysis^2^, n = 63** | |
| --- | --- | --- | --- | --- | --- | --- |
|  |  | **aIRR (95% CI)** | ***P*-value** |  | **aIRR (95% CI)** | ***P*-value** |
| Age (years) | |  | 0.428 |  |  | 0.643 |
|  | 18-24 | Reference |  |  | Reference |  |
|  | ≥25 | 0.81 (0.49-1.35) |  |  | 1.45 (0.30-7.02) |  |
| Total number of male partners, past 3 months | |  | 0.538 |  |  | 0.612 |
|  | None | 1.26 (0.55-2.88) |  |  | 1.12 (0.09-13.41) |  |
|  | One | Reference |  |  | Reference |  |
|  | Two or more | 0.72 (0.38-1.38) |  |  | 0.28 (0.03-2.06) |  |
|  | Missing response | ** |  |  | ** |  |
| Receptive anal intercourse, past 3 months | |  | 0.576 |  |  | 0.261 |
|  | No | Reference |  |  | Reference |  |
|  | Yes | 1.35 (0.80-2.29) |  |  | 4.62 (0.32-66.56) |  |
|  | Missing response | ** |  |  |  |  |
| Condom use during last sex | |  | 0.464 |  |  | 0.435 |
|  | No | Reference |  |  | Reference |  |
|  | Yes | 0.71 (0.41-1.22) |  |  | 0.46 (0.07-3.18) |  |
|  | Missing response | ** |  |  |  |  |
| Receiving payment for sex, past 3 months | |  | 0.191 |  |  | 0.611 |
|  | No | Reference |  |  | Reference |  |
|  | Yes | 0.60 (0.34-1.04) |  |  | 1.61 (0.26-9.94) |  |
|  | Missing response | ** |  |  |  |  |
| Self-reported daily oral PrEP use | |  | 0.112 |  |  | 0.346 |
|  | Off-PrEP | Reference |  |  | Reference |  |
|  | On-PrEP | 1.57 (0.89-2.76) |  |  | 2.46 (0.38-15.94) |  |
|  | Missing response | 5.0 (0.64-38.76) |  |  |  |  |
| CT/NG infection at any site at baseline^1^ | |  | **<0.001** |  |  | 0.962 |
|  | No | Reference |  |  | Reference |  |
|  | Yes | **3.27 (1.95-5.49)** |  |  | 1.05 (0.13-8.52) |  |

aIRR=Adjusted incidence rate ratio, CI=Confidence interval, CT=Chlamydia trachomatis, MSM=Men who have sex with men, PrEP=Pre-exposure prophylaxis, TGW=Transgender women.

^1^Participants (n=7) who tested positive CT/NG at baseline with no record of treatment were excluded from incidence analysis.

^2^Model of conceptualized confounders of the association between gender identity and prevalent or incident CT/NG including payment for sex, a priori rather than sexual partner type for last male and for last female partner, due to the conceptual overlap in these three variables.

**No incident infection among participants who had a ‘missing response’. aIRR (95% CI) = <0.00 (0.00-Not determined) or >100.00 (0.00-Not determined).

Bold values indicate variables whose 95% CI of the aIRR did not cross 1.
