# Supplementary material for: Chlamydia and gonorrhoea infections in young Kenyan HIV-negative cisgender men who have sex with men and transgender women: a multicentre cohort study
Source: BMJ Open. 2025 Aug 1;15(7):e098916. doi: 10.1136/bmjopen-2025-098916 (PMC12314943; doi:10.1136/bmjopen-2025-098916)
Supplement: Supplementary file 2 [file bmjopen-2025-098916.reviewer_comments.pdf]

# PEER REVIEW HISTORY

BMJ Open publishes all reviews undertaken for accepted manuscripts. Reviewers are asked to complete a checklist review form and are provided with free text boxes to elaborate on their assessment. These free text comments are reproduced below.

## ARTICLE DETAILS

### Title (Provisional)

Chlamydia and gonorrhoea infections in young Kenyan HIV-negative cisgender men who have sex with men and transgender women: a multicentre cohort study

### Authors

Sanders, Eduard J; Wahome, Elizabeth; Otieno, Fredrick; Kimani, Joshua; Bentzer, Alice; Okall, Duncan; Nzioka, Joseph; Gichuru, Evans; van der Elst, Elise M; Bailey, Robert C; Mehta, Supriya D.; Graham, Susan

---

## VERSION 1 - REVIEW

---

| | |
|---|---|
| **Reviewer** | **1** |
| **Name** | **Hamill, Matthew M** |
| **Affiliation** | **The Johns Hopkins University School of Medicine, Division of Infectious Diseases** |
| **Date** | **20-Mar-2025** |
| **COI** | **None** |

---

This manuscript is a very welcome addition to the limited data on 2 bacterial STIs (CT/NG) in MSM and TWG in sub Saharan Africa. It describes prevalent CT and NG in 570 MSM and 80 TGW and incident infection over 6 months in 2021-2022 during the COVID pandemic. That the authors take care to separately consider MSM and TGW is also welcome.

Overall, this analysis is interesting and well written. If the issues addressed below are addressed it would be an important addition to the epidemiology of curable STIs in MSM and TGW in Africa.

There are some major concerns enumerated below. Chief among them is the conclusion that sexual behavioral differences are driving differences when this is not demonstrated in the adjusted analyses. It also states that untreated infections in partners contributed to increased infection but there are no data to support this. Indeed, in the limitations section the authors state that that there was no record of outcomes of EPT.

Two factors that are very notable by their absence are data on HIV and syphilis. Specifically, were there HIV seroconversions throughout the study and were these related to prevalent or

incident CT/NG? In a manuscript that focuses heavily on STI chemoprevention it is noticeable that syphilis is not mentioned. The authors should include syphilis data or explain why these tests were not carried out.

Below are some major observations followed by minor ones.

Abstract:

Conclusion describes differences in CT/NG because of sexual exposure differences but does not give any sexual behavior data in the results. Also untreated infections in partners which is not in results. The conclusions are not supported by data in results and should be modified accordingly.

Intro:

Line 17. Seems to consider TGW as 'men'.

Methods:

Study procedures:

- Should indicate if urogenital samples were urine or vaginal swabs in TGW/ Also if all swabs were by self-collect or clinician collect.

- Need more information on how treatment adherence for CT treatment was assessed. The differences in incident STI seems largely driven by prevalent CT. It is possible that the incident CT was not new infection but persistence if adherence to high dose doxycycline was low.

Measures/covariates:

- Was recent antibiotic use data collected? At baseline and/or at follow up visits? This could influence both prevalence and incidence data. During COVID-19 pandemic especially, antimicrobials with anti-CT/NG activity (e.g., azithromycin) were commonly used.

Results:

- Were there any HIV seroconversions? And how did this relate to CT/NG. This is important to discuss either to say that there were none or to describe CT/NG status in that group.

- Study population. Needs to confirm how many had baseline OP testing and what proportion in MSM and TGW. Were there differences in proportions of MSM and TGW who received OP testing?

- Page 9, lines 54-56. The authors state that TGW had a 3x higher NG IR for each anatomical site. However, there was 0 incident cases of urogenital CT in TGW. I would modify the statement to say that differences were approx. 3x in extragenital but there were no urogenital incident infections in TGW.

- Page 10, line 17. It is an interesting finding that no participant with NG at baseline had evidence of a repeat infection. This could be made clearer rather than saying it 'was not noted for NG infections'.

- Can the authors explain why there were so many variables selected in the regression? The multivariable model includes several variables that were not significant in the bivariable model. There may be an explanation for their inclusion but it is not evident.

Discussion:

- Page 12, lines 33-36 state: Overall, differences in CT/NG prevalence and incidence between TGW and MSM in this cohort appear to be primarily related to differences in sexual exposure. However, this is not demonstrated in your data. There were no significant differences in behaviors in the multivariable model for either prevalent or incident CT/NG. Indeed, there were no significant bivariable sexual behavioral associations in the incidence data. It appears that the authors are relying on supplementary table 1 to explain behavioral differences in MSM and TGW as evidence of differences in CT/NG infection, though there are no clear differences in the multivariable models. If this is the case, it should be carefully justified.

This conclusion is not supported by the presented data in this analysis. Data from a previous analysis is important but should not be used to support one of the main conclusions of this analysis. This paragraph (and its corresponding section in the abstract should be revised).

- Page 14, line 22. The authors should clarify what relatively costly means in the Kenyan context. How much is 200mg compared with weekly wage? In many settings, generic doxycycline is considered very cheap in comparison with LA BPG to treat syphilis for example.

- Page 15, lines 36-45. Suggest adding to limitation section: no adherence monitoring of 7 days of doxycycline for CT therefore uncertain if incident CT is new or untreated from baseline visit. No NG AST data presented - possible that some reductions in MIC to cefixime in NG. Azithro was used to treat CT/NG coinfections - there are ample data that azithro is substantially less efficacious for rectal CT compared with doxy.

Minor issues:

Abstract - suggest consistency in number of decimals for p-values.

Study procedures:

- Line 26 implies all had triple site testing. Suggest that the authors state in the first sentence of 'study procedures' that participants were tested for genital & rectal CT/NG and that a subset were tested for OP infection.

- Please add ref for Kenyan STI treatment guidelines. I was unable to locate these to confirm the 200mg dose of doxycycline for CT which a higher dose than expected.

- Please confirm if EPT consisted of medications provided or a prescription provided.

Conceptualized confounders:

- Line 49. Why was paid for sex included but not paying for sex?

- Page 9, lines 3-8. Can the authors better describe the conceptual overlap between sex partner type and payment for sex so that only 1 was selected because both seem important.

- Justifying choosing the 6-month gap between baseline and repeat CT/NG sampling is important. Were the 3 monthly data not available or was there another reason. CDC for example recommend repeat testing at 3 months post-index STI diagnosis.

Discussion:

- Page 12, line 8. You state in young MSM & TGW. More than half were aged 25-29. Suggest changing in MSM/TGW aged <30 years. Mid-late 20s not what we think of as youth.

- Page 14, lines 28-42. It is somewhat unusual to highlight planned future research in the discussion at least in this level of detail. It is of course germane but distracts from the focus of the current analysis. Suggest reducing this.

- Page 15, lines 10-22. This section is a repetition of what is presented earlier in the discussion. Suggest consolidating these two areas.

---


| | |
|---|---|
| **Reviewer** | **2** |
| **Name** | **Flores, Juan** |
| **Affiliation** | **Universidad Privada San Juan Bautista, Escuela de Tecnología Médica** |
| **Date** | **27-Mar-2025** |
| **COI** | **None** |


---

The manuscript presents a valuable measurement focused on the context of HIV-negative individuals in Kenya and the identification of factors associated with CT/NG infection. While it is known that there is a significant difference in risk behavior between TSW (Transgender Women) and MSM (Men who have Sex with Men), the study has stratified this information into main and supplementary tables, providing a clearer understanding for readers.

Summary:

Line 45: Please clarify the type of participants being referred to (MSM/TGW).

Variable Measurement:

It is crucial to provide context regarding PrEP prophylaxis, as it is one of the main factors associated with the incidence of CT/NG. Specify whether it was taken orally or by injection. If administered orally, indicate whether it is known (consider this in the limitations section) if it was taken daily.

Results:

In the methods section (line 8), three sites are mentioned, with two located in Coastal Kenya; however, these two sites are not specified. This should be clarified at the beginning of Table 1 for better understanding.

Tables:

Ensure that the percentages of the relative frequencies sum to 100%. For instance, in Table 1 (gender of the last sexual partner).

In Tables 2, 4, and S2, please relocate the p-value values, as they are incorrectly placed on the same line as the reference.

Clarify whether the adjusted models consistently account for the confounders: (1) the type of sexual partner for the last male partner and the type of sexual partner for the last female partner, and (2) receiving payment for sex.

Discussion:

In paragraph 2, line 54, clarify that the conclusion pertains to CT/NG at any site and encompasses the entire studied population (MSM + TGW).

On page 2 of the discussion, line 5 needs to emphasize more evidence-based support. Review articles like this one to generate evidence-based explanations: https://pmc.ncbi.nlm.nih.gov/articles/PMC6447480/.

In the limitations section, it is also important to mention that, although the study was unable to sequence new cases for comparison with the baseline, this should be considered in future studies to determine whether they represent reinfections or antibiotic resistance. Since it has been stated that there has been no follow-up on medication adherence during the follow-up period, this could alter the interpretation that behaviors are the primary associated factors.

---

## VERSION 1 - AUTHOR RESPONSE

Reviewer: 1
Dr. Matthew M Hamill, The Johns Hopkins University School of Medicine
Comments to the Author:
This manuscript is a very welcome addition to the limited data on 2 bacterial STIs (CT/NG) in MSM and TWG in sub Saharan Africa. It describes prevalent CT and NG in 570 MSM and 80 TGW and incident infection over 6 months in 2021-2022 during the COVID pandemic. That the authors take care to separately consider MSM and TGW is also welcome. Overall, this analysis is interesting and well written. If the issues addressed below are addressed it would be an important addition to the epidemiology of curable STIs in MSM and TGW in Africa.

**We thank the reviewer for his positive comments.**

There are some major concerns enumerated below. Chief among them is the conclusion that sexual

behavioral differences are driving differences when this is not demonstrated in the adjusted analyses. It also states that untreated infections in partners contributed to increased infection but there are no data to support this. Indeed, in the limitations section the authors state that that there was no record of outcomes of EPT.

**We have reflected on Dr. Hamill's concern in regards to our original conclusion and discussion about the potential effect of untreated infections in partners. In response to the reviewer's concerns, we have edited our conclusion and omitted the statement that sexual behavioural differences were the driver of any differences in CT/NG infection between MSM and TGW. In regards to untreated infections, we added that "Untreated infections in partners have likely contributed to increased infection." We have also stressed the limitation that we had no record of EPT outcomes.**

Two factors that are very notable by their absence are data on HIV and syphilis. Specifically, were there HIV seroconversions throughout the study and were these related to prevalent or incident CT/NG? In a manuscript that focuses heavily on STI chemoprevention it is noticeable that syphilis is not mentioned. The authors should include syphilis data or explain why these tests were not carried out.

**CT/NG testing was an added objective to what was initially an HIV incidence cohort. While participants for the HIV incidence study were enrolled in the period 2019-2021, CT/NG testing was done at two time points in the period October 2021-December 2022. In the last period of the cohort study, HIV incidence was lower than expected (~1 per 100 person years) and only 3 HIV seroconversions were captured. We are therefore not able to correlate CT/NG infection with incident HIV infections. In response to this comment, we have added information on the limited number of HIV seroconversions that took place in the study period.**

**Regarding syphilis, its prevalence has been low in MSM in Kenya for many years. In a cross-sectional study among 486 MSM in coastal Kenya, syphilis prevalence was 3.3% (1.7-4.9; Grijsen et al, STI 2008). In an HIV incidence study among 449 enrolled MSM in the same location, syphilis prevalence was 1.6% (Sanders et al, AIDS, 2013). In response to the reviewer comment, we have added that syphilis testing was not conducted in this study.**

Below are some major observations followed by minor ones.

Abstract:
Conclusion describes differences in CT/NG because of sexual exposure differences but does not give any sexual behavior data in the results. Also untreated infections in partners which is not in results. The conclusions are not supported by data in results and should be modified accordingly.
**This has been done.**

Intro:
Line 17. Seems to consider TGW as 'men'.
**Thank you for this correction. We have changed "men" to "vulnerable populations."**

Methods:
Study procedures:
- Should indicate if urogenital samples were urine or vaginal swabs in TGW/ Also if all swabs were by self-collect or clinician collect.

**In our revised submission, we have clarified that all participants provided a urine sample and that research clinicians collected rectal or oropharyngeal swabs.**

- Need more information on how treatment adherence for CT treatment was assessed.

**We have clarified that while treatment with cefixime or azithromycin was directly observed, 7-day courses of doxycycline were provided to participants after education and instruction by research clinicians, without follow-up to confirm treatment completion. This is in line with standard care in Kenya but an acknowledged limitation of our research study.**

The differences in incident STI seems largely driven by prevalent CT. It is possible that the incident CT was not new infection but persistence if adherence to high dose doxycycline was low.
As above, we did not collect information on doxycycline adherence, so this is possible. **In addition, we were not able to ascertain if CT infections were cured in this study. We have added this as a limitation of the current study.**

Measures/covariates:
- Was recent antibiotic use data collected? At baseline and/or at follow up visits? This could influence both prevalence and incidence data. During COVID-19 pandemic especially, antimicrobials with anti-CT/NG activity (e.g., azithromycin) were commonly used.

**We did not capture recent antibiotic use in this study. Again, this is a limitation that we have now added to the discussion section.**

Results:
- Were there any HIV seroconversions? And how did this relate to CT/NG. This is important to discuss either to say that there were none or to describe CT/NG status in that group.

**As explained above, HIV incidence was low and CT/NG testing was added at the end of the cohort study. During the CT/NG study, there were a total of 3 HIV seroconversions (all MSM). We have added this information to the results section.**

Study population. Needs to confirm how many had baseline OP testing and what proportion in MSM and TGW. Were there differences in proportions of MSM and TGW who received OP testing?
**Oropharyngeal infections swabs were tested in a random (1/3) sample of participants. These included 202 (35%) MSM and 33 (41%) TGW (p=0.311).**

- Page 9, lines 54-56. The authors state that TGW had a 3x higher NG IR for each anatomical site. However, there was 0 incident cases of urogenital CT in TGW. I would modify the statement to say that differences were approx. 3x in extragenital but there were no urogenital incident infections in TGW.

**Thank you for pointing this out. We corrected our finding as follows: "While no urogenital incident infections occurred in TGW, TGW had an approximately 3-fold higher NG IR per 100 person-years relative to MSM for the combined rectal and oropharyngeal sites."**

- Page 10, line 17. It is an interesting finding that no participant with NG at baseline had evidence of a repeat infection. This could be made clearer rather than saying it 'was not noted for NG infections'.

**We changed the text as follows: "This difference was driven by repeat CT infections, as no new NG infections occurred in participants with an NG infection at baseline (Table 3)."**

- Can the authors explain why there were so many variables selected in the regression? The multivariable model includes several variables that were not significant in the bivariable model. There may be an explanation for their inclusion but it is not evident.

**As mentioned in our data analysis section, we had data on many conceptualized confounders of the association between gender identity and prevalent or incident CT/NG, including age, condom use for RAI/IAI, condom use during last sex, receptive anal intercourse, insertive anal intercourse, sexual partner type for the last male and last female partner reported, number of male and female partners, receiving payment for sex, and PrEP use. These potential confounders were assessed for multicollinearity using variance inflation factor (VIF), with only one variable included in the model when a pair of potential confounders had a VIF of 5 or more.**

**After creating an initial model with all potential confounders included, we selected the most parsimonious model (i.e., the model with the lowest Bayesian Information Criterion) for each outcome. [1] This approach seeks to avoid overfitting by introducing a penalty term for the number of parameters in the model. We adopted this approach rather than a model selection approach based on P values, which are not designed for measuring the goodness of model fit and cannot provide any evidence in favor of a candidate model versus another model.**

Discussion:

- Page 12, lines 33-36 state: Overall, differences in CT/NG prevalence and incidence between TGW and MSM in this cohort appear to be primarily related to differences in sexual exposure. However, this is not demonstrated in your data. There were no significant differences in behaviors in the multivariable model for either prevalent or incident CT/NG. Indeed, there were no significant bivariable sexual behavioral associations in the incidence data. It appears that the authors are relying on supplementary table 1 to explain behavioral differences in MSM and TGW as evidence of differences in CT/NG infection, though there are no clear differences in the multivariable models. If this is the case, it should be carefully justified. This conclusion is not supported by the presented data in this analysis. Data from a previous analysis is important but should not be used to support one of the main conclusions of this analysis. This paragraph (and its corresponding section in the abstract should be revised).

**The reviewer is correct. We have changed our abstract conclusion and discussion sentence and removed the overemphasis on sexual exposure differences.**

- Page 14, line 22. The authors should clarify what relatively costly means in the Kenyan context. How much is 200mg compared with weekly wage? In many settings, generic doxycycline is considered very cheap in comparison with LA BPG to treat syphilis for example.

**We have expanded upon this in the revised manuscript: "It should be noted that regular use of doxyPEP is relatively costly compared to infrequent syndromic treatment as needed, leads to increased antibiotic consumption, and may induce resistance in non-STI pathogens. In Kenya, treatment of syphilis and syndromic STI is provided free of charge to patients, whereas doxyPEP is currently unavailable unless patients pay out of pocket."**

- Page 15, lines 36-45. Suggest adding to limitation section: no adherence monitoring of 7 days of doxycycline for CT therefore uncertain if incident CT is new or untreated from baseline visit. No NG AST data presented - possible that some reductions in MIC to cefixime in NG. Azithro was used to treat CT/NG coinfections - there are ample data that azithro is substantially less efficacious for rectal CT compared with doxy.

**In the revised manuscript, we have mentioned these limitations: no data on doxycycline adherence, no antimicrobial sensitivity testing, and the treatment of combined CT/NG infections with cefixime and azithromycin rather than ceftriaxone and doxycycline.**

Minor issues:
Abstract - suggest consistency in number of decimals for p-values.
**We now include 3 digits after the decimal for each p-value in the abstract.**

Study procedures:
- Line 26 implies all had triple site testing. Suggest that the authors state in the first sentence of 'study procedures' that participants were tested for genital & rectal CT/NG and that a subset were tested for OP infection.

**We have done this.**

- Please add ref for Kenyan STI treatment guidelines. I was unable to locate these to confirm the 200mg dose of doxycycline for CT which a higher dose than expected.

**We have added the reference for the Kenyan STI treatment guidelines. The dose of doxycycline was incorrectly worded – it should have been 100 mg twice daily. This has been corrected and citation added.[2]**

- Please confirm if EPT consisted of medications provided or a prescription provided.

**In the revision, we have clarified that medication was provided.**

Conceptualized confounders:
- Line 49. Why was paid for sex included but not paying for sex?

**While paying for sex is likely associated with increased risk of STI, this risk likely runs through insertive anal intercourse and condom use for insertive anal intercourse. We have repeatedly found that being paid for sex is more risky, as it usually involves unprotected receptive anal intercourse with multiple partners. In response to this question, we have added paying for sex to modelling, presenting results in the bivariable analysis column. Paying for sex was not included in any of the final multivariable models based on BIC variable selection.**

- Page 9, lines 3-8. Can the authors better describe the conceptual overlap between sex partner type and payment for sex so that only 1 was selected because both seem important.

**For report of the sex partner type, paying/paid was one option. Participants who reported receiving payment for sex in the past 3 months were significantly more likely to report that their last partner had been paying/paid. In addition, those who reported receiving payment for sex in the past 3 months had higher numbers of partners. We had challenges fitting models in which the last sex partner type were included due to non-convergence, so elected to omit the last sex partner type variable and include only variables that captured partner numbers and receiving payment for sex in modelling. We have clarified this in the revised manuscript.**

- Justifying choosing the 6-month gap between baseline and repeat CT/NG sampling is important. Were the 3 monthly data not available or was there another reason. CDC for example recommend repeat testing at 3 months post-index STI diagnosis.

**Because we had only limited funding for NAAT-based testing, we had to be strategic. NAAT testing is currently not standard of care in Kenya and is unlikely to**

**become so in the near future because of its cost, even if only offered every 6 or 12 months. We decided to test at baseline and 6 months in order to capture incident cases over a time period that might be more feasible for programmatic screening of MSM in this setting. We have added a brief justification to the manuscript text.**

Discussion:
- Page 12, line 8. You state in young MSM & TGW. More than half were aged 25-29. Suggest changing in MSM/TGW aged <30 years. Mid-late 20s not what we think of as youth.
**We have changed the text to refer to 18- to 29-year-old MSM and TGW.**

- Page 14, lines 28-42. It is somewhat unusual to highlight planned future research in the discussion at least in this level of detail. It is of course germane but distracts from the focus of the current analysis. Suggest reducing this.
**We have deleted this given uncertainty in continued funding from the NIH to conduct this trial.**

- Page 15, lines 10-22. This section is a repetition of what is presented earlier in the discussion. Suggest consolidating these two areas.
**We have cut these sentences in the revised manuscript, as it is true that the points about asymptomatic infections and the cost of screening are made elsewhere in the discussion.**

Reviewer: 2
Mr. Juan Flores, Universidad Privada San Juan Bautista
Comments to the Author:
The manuscript presents a valuable measurement focused on the context of HIV-negative individuals in Kenya and the identification of factors associated with CT/NG infection. While it is known that there is a significant difference in risk behavior between TSW (Transgender Women) and MSM (Men who have Sex with Men), the study has stratified this information into main and supplementary tables, providing a clearer understanding for readers.
**We thank the reviewer for this comment.**

Summary:
Line 45: Please clarify the type of participants being referred to (MSM/TGW).
**We believe this comment pertains to the abstract, where we mention that incident CT/NG infection was increased among participants with baseline CT/NG infection. Both gender identity and baseline CT/NG infection were independent predictors in this model, which included all participants (both MSM and TGW). We are therefore referring to all participants (both MSM and TGW).**

Variable Measurement:
It is crucial to provide context regarding PrEP prophylaxis, as it is one of the main factors associated with the incidence of CT/NG. Specify whether it was taken orally or by injection. If administered orally, indicate whether it is known (consider this in the limitations section) if it was taken daily.
**In Kenya, only oral PrEP is currently available. While intermittent PrEP is acknowledged in guidelines, daily PrEP is recommended for populations at high risk for HIV. We have clarified that we are referring to daily oral PrEP.**

Results:
In the methods section (line 8), three sites are mentioned, with two located in Coastal Kenya; however, these two sites are not specified. This should be clarified at the beginning of Table 1 for

better understanding.

**Thank you for this feedback. We now specify in the methods text that the two coastal Kenya sites were in Mtwapa and Malindi.**

Tables:
Ensure that the percentages of the relative frequencies sum to 100%. For instance, in Table 1 (gender of the last sexual partner).

**We have corrected this problem with Table 1, which did not always include the number and percentage of missing observations.**

In Tables 2, 4, and S2, please relocate the p-value values, as they are incorrectly placed on the same line as the reference.

**This has been done.**

Clarify whether the adjusted models consistently account for the confounders: (1) the type of sexual partner for the last male partner and the type of sexual partner for the last female partner, and (2) receiving payment for sex.

**We apologize for the lack of clarity in our description of the modelling. We included receiving payment for sex, rather than sexual partner type, in all multivariable models due to due to the conceptual overlap when the three variables were included in modelling.**

Discussion:
In paragraph 2, line 54, clarify that the conclusion pertains to CT/NG at any site and encompasses the entire studied population (MSM + TGW).

**This has been done.**

On page 2 of the discussion, line 5 needs to emphasize more evidence-based support. Review articles like this one to generate evidence-based explanations:
https://pmc.ncbi.nlm.nih.gov/articles/PMC6447480

**We have revised this paragraph in response to comments from reviewer 1. We did not incorporate the article recommended here, as it refers to repeat chlamydia infections in women and the possibility of immunity leading to lower bacterial loads, which we did not measure in our study**.

In the limitations section, it is also important to mention that, although the study was unable to sequence new cases for comparison with the baseline, this should be considered in future studies to determine whether they represent reinfections or antibiotic resistance. Since it has been stated that there has been no follow-up on medication adherence during the follow-up period, this could alter the interpretation that behaviors are the primary associated factors.

**In response to feedback from both reviewers, we have changed our conclusion that exposure differences are driving differences in infection rates and provided additional information on limitations of the study that preclude further investigation of the cause of repeat infections in this study.**

Citations added:

1. Raferty AE. Bayesian Model Selection in Social Research. Sociological Methodology 1995; 25: 111-63.

2. MoH. Kenya National Guidelines for Prevention, Management and Control of Sexually Transmitted Infections. Nairobi, Kenya, 2018.

---

## VERSION 2 - REVIEW

---

**Reviewer** 1

**Name** Hamill, Matthew M

**Affiliation** The Johns Hopkins University School of Medicine, Division of Infectious Diseases

**Date** 24-Jun-2025

**COI**

---

I thank the authors for their revisions. The resulting manuscript is stronger as a result.
